# Mitophagy in spermatogenesis: Roles and mechanisms

**DOI:** 10.1016/j.bbrep.2024.101730

**Published:** 2024-05-11

**Authors:** Nabil Eid, Md Abdul Alim Al-Bari

**Affiliations:** Anatomy Department, Division of Human Biology, School of Medicine, International Medical University (IMU University), 57000, Kula Lumpur, Malaysia; Department of Pharmacy, University of Rajshahi, Rajshahi, 6205, Bangladesh

We read with interest the review article “Mitophagy and Spermatogenesis: Role and Mechanisms” recently published in Biochemistry and Biophysics Reports [1]. In this review, the authors summarized the various mechanisms of conventional mitophagy and the roles of mitophagy in spermatogenesis, citing our paper [2]. In this letter, we highlight several issues related to this review [1], which need clarification and correction.

In the abstract, the authors mentioned that: Furthermore, studies have shown that inhibited autophagy-infected spermatozoa had reduced motility and increased amounts of phosphorylated PINK1, TOM20, caspase 3/7, and AMP [1]. This statement is not correct. I think they mean that inhibition of autophagy in spermatozoa results in reduced motility and increased amounts of PINK1, TOMO20, caspases 3 and 7 and AMP. This is supported by a recent study in human spermatozoa. In this study, the authors found that activation of autophagy in human spermatozoa resulted in a significant increase in motility and a decrease in PINK1 (sensor of mitochondrial damage and initiator of mitophagy), TOM20 (mitochondrial marker) expression and caspase 3/7 (apoptotic markers) activation. Conversely, autophagy inhibition resulted in decreased sperm motility and viability, which was associated with increased PINK1, TOM20, caspase 3/7 expression levels and AMPK phosphorylation. They concluded that selective autophagy of damaged mitochondria (mitophagy) in sperm functions to improve motility and viability and suppress apoptosis, thus enhancing fertility [3].

There are several truncated sentences without meaning, such as: This process is collectively referred to as LC3, in page 3, left lower paragraph. The microtubule-associated protein 1A/1B light chain 3 (LC3) is the principal marker of autophagy. LC3-II form is responsible for formation of autophagosomes engulfing damaged mitochondria (mitophagosomes). These mitophagosomes are characterized by accumulations of PINK1 and Parkin on the outer mitochondrial membrane (OMM). They fuse with lysosomes forming mitolysosomes, which are degraded by lysosomal cathepsins [2]. In the same page (lower right paragraph), they also mentioned that: However, incorrect phagocytotic cell activities lead to spermatid mortality due to the inability to modify and remove undesirable cellular contents. I think they mean that autophagic not phagocytic [1,4]. Phagocytosis is the removal of dead cells by specific phagocytes. In our recent study, using light and electron microscopic methods, we showed the enhanced testicular germ cell apoptosis in ethanol-treated rats. These apoptotic germ cells including the sperm were found to be cleared by the phagocytic Sertoli cells [5].

The authors discussed the various mechanisms of canonical mitophagy mainly, the PINK1-Parkin pathway and receptor-related such as BNIP3L/NIX [1]. These mechanisms can clear the whole damaged mitochondrion via mitophagy. However, there are several non-canonical mitophagy mechanisms such as Parkin-mediated mitochondrial-derived vesicles which can eliminate only fragments of mitochondrion [6,7]. Moreover, mitochondria can be removed from cells via exospheres (extracellular vesicles containing mitochondria) formed by cytoplasmic membrane budding [6].

Although the authors demonstrated a figure showing the mechanisms of mitophagy [1], this figure did not show the various mechanisms of conventional mitophagy as shown in Fig. 1, republished from our recent review [8].

**Fig. 1:**
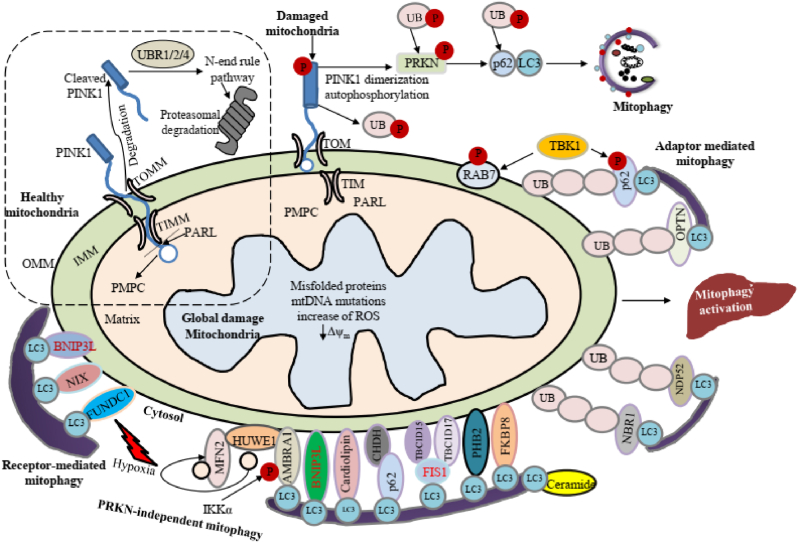
Mechanisms of Parkin dependent and independent mitophagy in mammalian cell. In healthy mitochondria, PINK1 is cleaved at the outer mitochondrial membrane (OMM) and degraded in the cytoplasm via the proteasome. On the other hand, upon mitochondrial damage, PINK1 accumulates on the OMM, stimulating Parkin translocation from cytoplasm to OMM, and subsequent removal of damaged or dysfunctional mitochondria via LC3-mediated mitophagy. Non-Parkin dependent mitophagy mechanisms include FUNDC1, BNIP3 and BNIP3L receptors which stimulate LC3-mediated mitophagy through LIR domains. In addition, PHB2, CL and BCL2L13 interact with LC3 and act as mitophagy receptors. Ceramide can act as a specific receptor for mitophagy by directly interacting with LC3 [8].

In a mature mammalian spermatozoon, approximately 72–80 mitochondria are present in the midpiece that can provide energy via oxidative phosphorylation for sperm cells [9]. Leydig cell mitochondria present lamellar cristae in close association with a gap between apposing lamellae, and the Leydig cell steroidogenesis is reliant on the functional mitochondria [[10], [11], [12]]. In addition, Cadmium-induced apoptosis of Leydig cells is mediated by excessive mitochondrial fission and inhibition of mitophagy, indicating the prosurvival role of mitophagy in Leydig cells [13].

Importantly, in the last paragraph before conclusions, citing our paper, the authors mentioned that using transmission electron microscopy (TEM), the authors showed that skeletal muscle cells of rats treated with ethanol had higher levels of mitochondrial damage [1]. However, we used TEM to show the enhanced mitochondrial damage and mitophagy in Sertoli cells of ethanol-treated rats. This enhanced mitophagy in Sertoli cells (the main supportive cell of spermatogenesis) has been found to be mediated by PINK-1-Parkin pathway as anti-apoptotic mechanism, suppressing the inflammatory response related to mitochondrial damage, and maintaining testicular homeostasis. In addition, enhanced Sertoli cell mitophagy may provide lactate for germ cells via a Warburg-like effect via catabolism of damaged mitochondria [2,5].

Finally, Overall, we hope that our comments will add to the work of the authors. Also, we hope that this letter would not be viewed as a negative comment on their review [1]. We do believe that our comments could be of interest to the readers of BB Reports.

## CRediT authorship contribution statement

**Nabil Eid:** Writing – review & editing. **Md Abdul Alim Al-Bari:** Writing – review & editing.

## Declaration of competing interest

Nothing to be disclosed regarding any potential competing or non-financial interests on behalf of all authors of the manuscript. This page includes a tool that authors can use to generate their declaration of interest.
